# Perceptions of water insecurity from urban and peri-urban Haiti: A quantitative analysis

**DOI:** 10.1371/journal.pone.0214789

**Published:** 2019-04-24

**Authors:** Elizabeth A. Wood, Hannah Douglas, Andrew J. Fiore, Robinson Bernier, Kelly S. Chapman

**Affiliations:** 1 Department of Environmental & Global Health College of Public Health and Health Professions University of Florida, Gainesville, FL, United States of America; 2 College of Public Health and Health Professions University of Florida, Gainesville, FL, United States of America; 3 Department of Anthropology University of Florida, Gainesville, FL, United States of America; University of Dhaka, BANGLADESH

## Abstract

Safe drinking water access has continued to be a growing issue in Haiti. Water accessibility, availability, and quality can have severe implications on health and safety, with those in urban areas often having more access. Key differences relating to water accessibility can be seen between the urban and peri-urban areas of Haiti. One major objective of this research is to examine the disparities between the two areas and determine limiting and enabling factors that are contributing to the perceived access to clean water. A cross-cultural household water insecurity experiences (HWISE) survey (n = 499) was distributed to determine barriers and accessibility to sufficient water quality and quantity at the household level. This paper explores the relationship between water insecurity between two urban and peri-urban communes in Haiti using this data. Fisher’s Exact and Kruskal-Wallis tests were used to identify significant differences between strata, and logistic regression was used to determine significant associations with water security outcomes. Results indicated there were differences in both the costs and the sources of drinking and non-drinking water between urban and peri-urban Haiti. Certain demographic and behavioral characteristics were associated with increased water insecurity, including a household size greater than five and experiencing injury during collection.

## Introduction

Haiti is a small country in the Caribbean with limited natural resources and is currently ranked as one of the poorest nations in the Western Hemisphere [1]. Additionally, it has the lowest improved water and sanitation coverage in the region, which directly impacts human health, environmental health, and economic stability [2–3]. Access to safe drinking water continues to be a growing issue in Haiti, more so after the damaging earthquake in 2010 and the constant hurricane damages that the region suffers. Water and sanitation conditions following the 7.0 magnitude earthquake in 2010 that struck Léogâne worsened largely due to the destruction of property, such as community and individual water sources. At the time of the earthquake, Haiti was unprepared for natural disasters due to the lack of adequate infrastructure and was not successful at preparing for the subsequent cholera endemic and the state of emergency that followed [4]. Following the reintroduction of cholera in Haiti, many governmental, non-governmental organizations, and other international organizations committed significant aid to improve health and infrastructure [5]. However, money that had been earmarked for improving water and sanitation was spent inefficiently due to an overwhelming lack of government capacity and support, excessive aid dependency, and lack of communication and consensus between Haitian political figures, Haitian Parliament, and the international community [6].

Water accessibility, availability, and quality has major implications on health and safety, with those in urban areas often having more access. Water insecurity has specifically been defined as having sufficient access or supply of safe water for daily function [7–9]. Through joint efforts, the World Health Organization (WHO) and the United Nations Children’s Fund (UNICEF) monitor global drinking water, sanitation and hygiene through the Joint Monitoring Programme (JMP). The JMP provides country-specific and world reports on water and sanitation coverage. In 2015, there were 522 million people worldwide that still use unimproved sources of drinking water and surface water, the two poorest drinking water sources [10]. According to the JMP report specifically for Haiti, urban areas have over twice the access to basic service water sources, where most people in rural areas collect water from unimproved sources [11]. Rural areas of Haiti have considerably less access to improved water sources compared to urban areas overall which ultimately contributes to disparities in health outcomes [2].

Key differences can be seen between the urban and rural areas; however, this study focuses primarily on the differences between urban and peri-urban. The two communities included in this study are Léogâne, located approximately 29 kilometers west of the capital, Port-au-Prince, and its neighboring commune, Gressier. While Léogâne has an estimated population of 200,000 people and is commonly viewed as urban, Gressier is much smaller in population with estimates ranging from 36,000 to as many as 75,000 people depending on the source and is considered peri-urban [12–13]. These communities were chosen as a result of their differences in water sources as a result of the 2010 earthquake. The main objective of this research is to examine the disparities between the two communes and determine limiting and enabling factors that are contributing to the perceived access to clean water. This study explores the circumstances surrounding water knowledge, attitudes, and practices at the household level in urban and peri-urban Haiti.

## Methods

### HWISE score

The HWISE survey is a validated, twenty-nine item instrument that measures water insecurity at the household level [14]. Questions ask about personal experiences with water in the past four weeks, for example “In the last four weeks, how frequently did you or anyone in your household worry that you would not have enough water for all your household needs?” Responses for each question included “Never”, “Rarely”, “Sometimes”, “Often”, and “Always”, which were scored zero through four, respectively. Those who responded “I don’t know” or refused were assigned missing values. The total HWISE score is a sum of the twenty-nine scored questions, and ranges from 0 to 116; favoring those with lower HWISE scores.

### Study design and sample

The University of Florida (UF) faculty and students conducted a cross-cultural household water insecurity experiences (HWISE) survey to determine barriers and accessibility to sufficient water quality and quantity at the household level in urban and peri-urban Haiti. The purpose of conducting this survey was to establish a comparable baseline of water access and behavior in Gressier and Léogâne, Haiti. The survey was distributed by randomly selecting households within a grid that overlaid Gressier and Léogâne. For houses that were empty or did not want to participate, a dice app was used to determine how many houses should be skipped before continuing. The surveys were conducted by a team of local Haitians that spoke fluent English, Creole, and French, and had training in enumeration and REDCap survey software. Tablets were used to collect survey data and GPS points using REDCap software, and a voice recorder app was used to record each conversation. Prior to each survey, the enumerator read from the oral consent form outlining the project and the voluntary status of each participant. The surveys were recorded for full transcription and validation of the information captured.

### Data analyses

Descriptive statistics were used to quantify the detailed distribution of sociodemographic and water characteristics; categories were then combined into most-similar groupings a priori. Fisher’s Exact tests identified significant differences in characteristic between Gressier and Léogâne; significant two-by-two and three-by-two tests both indicate that the proportions of a variable differ between locations. Numeric measures were generally found to be non-normally distributed, therefore the Kruskall-Wallis test was used to identify significant differences in numeric measures between location and stratified water sources. Logistic regression was conducted to determine odds significantly associated with water characteristics. Variables were selected forward stepwise from a full model of socio-demographic and behavioral characteristics using Akaike’s Information Criterion (AIC), a measurement that reduces fitting error while penalizing models with too many parameters [15]. Missing data were multiply imputed using the R package “mice” [16]; all analyses were conducted in R version 3.5.1.

### Normative income, education, safe work, and safe water

A component of the HWISE survey asks respondents to place themselves on a rung of a “ladder” corresponding to their standing in the community in realms of income, education, safe work, and safe water. This rung is translated to a rating ten through one, with ten indicating low self-perception. These data were dichotomized into “low rung” and “high rung” if a respondent indicated above or below the community’s 50^th^ percentile (median), which is identified in Table 1.

**Table 1:** Comparison of participant characteristics (Fisher’s Exact test).

	n (%)		Fisher’s Exact test	
	Gressier 295 (59.1)	Léogâne 204 (40.9)	P	OR (95% CI)
House type			0.3244	0.60 (0.19, 1.80)
Owned or rented house	287 (97.3)	195 (95.6)		
Other residence	8 (2.7)	9 (4.4)		
Occupation			0.5854	1.11 (0.76, 1.61)
Employed	153 (51.9)	111 (54.4)		
Not employed, homemaker, student	142 (48.1)	93 (45.6)		
Works in NGO sector			0.6002	1.18 (0.68, 2.02)
Yes	39 (13.2)	31 (15.2)		
No	256 (86.8)	173 (84.8)		
Works in healthcare sector			0.7621	0.87 (0.45, 1.65)
Yes	31 (10.5)	19 (9.3)		
No	256 (89.5)	173 (90.7)		
Education about health			0.0175	0.64 (0.44, 0.94)
Received	148 (50.2)	80 (39.2)		
Not received	174 (49.8)	124 (60.8)		
Education from work			0.2578	0.76 (0.47, 1.22)
Received	65 (22.0)	36 (17.7)		
Not received	230 (78.0)	168 (82.3)		
Drinking water source			0.0045	-
Pipe, pump, or well	145 (49.3)	81 (40.7)		
Vendor, person, truck, bottled, or sachet	138 (46.9)	117 (58.8)		
Rain, surface water, or other	11 (3.7)	1 (0.5)		
Non-drinking water source			< 0.001	-
Pipe, pump, or well	226 (77.4)	188 (91.9)		
Vendor, person, truck, bottled, or sachet	45 (15.4)	4 (2.0)		
Rain, surface water, or other	21 (7.2)	5 (2.5)		
Drinking and non-drinking water sources			< 0.001	0.49 (0.32, 0.73)
Are the same	125 (43.0)	53 (26.9)		
Are different	166 (57.0)	144 (73.1)		

### Ethics

Fieldwork for this study was completed from February to June 2018. The University of Florida Institutional Review Board approved this study October 31, 2017 (IRB201702549). The Haitian Ministry of Health ethics board also approved this study on February 22, 2018.

## Results

### Participants

In total, there were 499 surveys that were analyzed, 295 were from the peri-urban commune and 204 were from a very concentrated area of the urban commune. The urban site, Léogâne, had fewer participants despite being more populated than Gressier largely because researchers wanted to focus on the more densely populated areas of the city. The average age of participants was approximately 36 years old in both communes, with roughly two adults and three children per household. Most participants in both Léogâne and Gressier lived in a house, although there are differences in whether the house is owned versus rented (Table 1). Other dwellings included apartments, farms, squatter community, refugee/internally displaced camp, or other. Occupations ranged from trading, education, and food service to being supported by family members within Haiti or abroad. Individual occupations are distributed differently between Gressier and Léogâne. About 15% of respondents from both locations work in the non-governmental organization sector, and about 10% work in the healthcare sector. Finally, 50% of respondents from Gressier report receiving education about health, compared to 39% of respondents in Léogâne, and about 20% of respondents from both locations have received health education from work.

A potentially relevant difference between the communes (11%) is that respondents in Gressier received information about health at significantly higher rates than those in Léogâne. At the time the survey was given, respondents in Léogâne paid more for water in the past month than those in Gressier. Additionally, there is a significant difference in the distribution of water sources between Gressier and Léogâne. As shown in Table 1, respondents from Gressier report higher proportions of drinking water sourced from a pipe, pump, or well and lower proportions from rain, surface water, or other methods.

### Water use characteristics

The mean Household Water Insecurity Security Experience score in Gressier is approximately 18, and in Léogâne roughly 20 (Table 1). The cost of water (using the local currency, Haitian Gourdes—HTG) in Gressier and Léogâne is distributed differently, with the maximum cost in Gressier being 7000 HTG, compared to 3200 HTG in Léogâne, and median cost being 0 HTG and 100 HTG. Respondents in both Gressier and Léogâne source their drinking and non-drinking water from a range of sources, though Gressier has more diversity among non-drinking sources than Léogâne.

There are significant differences in the distribution of drinking and non-drinking water sources between Gressier and Léogâne. Specifically, a greater proportion of respondents in Gressier source their drinking water from rain, surface water, or other sources. Further, an overwhelming majority of respondents in Léogâne source their non-drinking water from a pipe, pump, or well, compared to a lower proportion of those in Gressier. There is also a significant difference in the proportion of individuals who get their drinking and non-drinking water sources from the same location.

When comparing numeric measures across drinking and non-drinking water sources in both locations, there are significant differences in the cost of water for protected or unprotected wells and rain, surface water, or other sources. Léogâne respondents who source their non-drinking water from a protected or unprotected well pay a median of 150 HTG. This is compared to drinking and non-drinking residents in Gressier and drinking residents in Léogâne, who pay a median of 0 HTG for protected or unprotected well water (Table 2).

**Table 2:** Differences in numeric measures between drinking and non-drinking sources and locations (Kruskall-Wallis).

		Gressier		Léogâne		Kruskal-Wallis X^2^	P Value
House or yard pipe							
		Drinking	Non-drinking	Drinking	Non-drinking		
Median	HWISE score	14.0	14.0	17.0	9.0	5.42	0.1435
	Cost of all water (HTG)	0.0	55.0	30.0	55.0	1.53	0.6747
	Household Size	5.0	5.0	6.0	6.0	8.75	0.0328
Hand pump							
		Drinking	Non-drinking	Drinking	Non-drinking		
Median	HWISE score	12.5	11.0	22.5	21.0	6.90	0.0752
	Cost of all water (HTG)	0.0	0.0	47.0	95.0	7.39	0.0604
	Household Size	5.0	5.0	5.0	5.0	1.84	0.6057
Protected or unprotected well							
		Drinking	Non-drinking	Drinking	Non-drinking		
Median	HWISE score	24.0	14.0	19.0	13.0	0.48	0.9225
	Cost of all water (HTG)	0.0	0.0	0.0	150.0	24.1	< 0.001
	Household Size	5.5	5.0	3.5	5.0	0.88	0.8282
Small vendor or another person							
		Drinking	Non-drinking	Drinking	Non-drinking		
Median	HWISE score	21.0	15.0	10.5	26.0	3.06	0.3827
	Cost of all water (HTG)	270.0	600.0	450.0	15.0	7.29	0.0632
	Household Size	5.0	5.0	5.0	4.0	1.08	0.7814
Truck							
		Drinking	Non-drinking	Drinking	Non-drinking		
Median	HWISE score	15.0	14.5	10	NA	0.13	0.9377
	Cost of all water (HTG)	250.0	200.0	100.0	NA	0.36	0.8347
	Household Size	3.0	4.0	5.5	NA	2.91	0.2340
		Gressier		Léogâne		Kruskal-Wallis X^2^	P Value
Bottle or sachet							
		Drinking	Non-drinking	Drinking	Non-drinking		
Median	HWISE score	13.0	13.5	14.0	NA	1.09	0.5804
	Cost of all water (HTG)	0.0	150.0	150.0	NA	6.14	0.0463
	Household Size	4.0	3.5	5.0	NA	8.83	0.0121
Rain, surface water, or other							
		Drinking	Non-drinking	Drinking	Non-drinking		
Median	HWISE score	10.0	24.0	5.0	18.0	6.99	0.0721
	Cost of all water (HTG)	0.0	275.0	0.0	35.0	6.89	0.0752
	Household Size	4.0	5.0	3.0	5.0	5.12	0.1630

When examining differences in numeric measures between water sources in the same location, there are significant differences in the cost of water by source. The cost of water significantly varies by source in Gressier and Léogâne drinking water and Gressier non-drinking water. The greatest median cost for a drinking source was 450 HTG for water from a small vendor or other person in Léogâne. The greatest median cost for non-drinking water was 600 HTG for water from a small vendor or other person (Table 3).

**Table 3:** Differences in numeric measures between water sources (Kruskall-Wallis test).

Primary drinking water sources								
	Gressier				Léogâne			
	Median							
	n (%)	HWISE score	Cost of all water*	Household Size	n (%)	HWISE score	Cost of all water*	Household Size
House or yard pipe	93 (31.6)	14.0	0.0	5.0	19 (9.5)	17.0	30.0	6.0
Hand pump	26 (8.8)	12.5	0.0	5.0	44 (22.1)	22.5	47.5	5.0
Protected or unprotected well	26 (8.8)	24.0	0.0	5.5	18 (9.0)	19.0	0.0	3.5
Small vendor or another person	43 (14.6)	21.0	270.0	5.0	24 (12.1)	10.5	450.0	5.0
Truck	22 (7.5)	15.0	250.0	3.0	4 (2.0)	10.0	100.0	5.5
Bottle or sachet	73 (24.8)	13.0	0.0	4.0	89 (44.7)	14.0	150.0	5.0
Rain, surface water, or other	11 (3.7)	10.0	0.0	4.0	1 (0.5)	5.0	0	3.0
All	295 (100)	14.0	0.0	5.0	204 (100)	14.0	100.0	5.0
Kruskal-Wallis X^2^		5.65	27.54	6.88		4.99	13.57	6.91
P Value		0.4639	< 0.001	0.3312		0.5456	0.0349	0.3293
Primary non-drinking water sources								
	Gressier				Léogâne			
	Median							
	n (%)	HWISE score	Cost of all water*	Household Size	n (%)	HWISE score	Cost of all water*	Household Size
House or yard pipe	93 (31.8)	14.0	55.0	5.0	21 (10.7)	9.0	55.0	6.0
Hand pump	50 (17.1)	11.0	0.0	5.0	72 (36.5)	21.0	95.0	5.0
Protected or unprotected well	83 (28.4)	14.0	0.0	5.0	95 (48.2)	13.0	150.0	5.0
Small vendor or another person	3 (1.0)	15.0	600.0	5.0	4 (2.0)	26.0	15.0	4.0
Truck	30 (10.3)	14.5	200.0	4.0	0 (0.0)	NA	NA	NA
Bottle or sachet	12 (4.1)	13.5	150.0	3.5	0 (0.0)	NA	NA	NA
Rain, surface water, or other	21 (7.2)	24.0	275.0	5.0	5 (2.5)	18.0	35.0	5.0
All	295 (100)	14.0	HTG 0.0	5.0	204 (100)	14.0	HTG 100.0	5.0
Kruskal-Wallis X^2^		8.73	19.40	6.74		6.92	8.96	2.54
P Value		0.1893	0.0036	0.3453		0.1402	0.0622	0.6381

*HTG

In Gressier, buying water was associated with sourcing non-drinking water from a pipe (OR = 3.09), and older age (OR = 1.65), but those who sourced drinking water from a pipe were at lower odds of buying water (OR = 0.38). In Léogâne, buying water was associated with experiencing past-year water shortage (OR = 1.27) and longer water collection times (OR = 1.17). However, respondents who reported high normative safe work, that they were responsible for their household’s water, and who worked were at lower odds of buying their water (OR = 0.35, 0.46, 0.30, respectively). In Gressier, factors associated with sourcing drinking water from a pipe in Gressier was sourcing non-drinking water from a pipe, greater household size, working (OR = 50.25, 2.17, 1.72, respectively). In Léogâne, factors associated with sourcing drinking water from a pipe include longer water collection time (OR = 4.32) and greater household size (OR = 3.12), but receiving health education, buying water, and rating a high normative safe work were associated with decreased odds of sourcing drinking water from a pipe (OR = 0.23, 0.26, 0.19, respectively).

When considering past-year water shortage in Gressier, larger household size, longer water collection, water storage in the home, and rating high normative safe water were associated factors (OR = 2.38, 2.20, 2.12, 2.01, respectively), with high normative income being associated with lower odds (OR = 0.48). In Léogâne, past-year water shortage was associated with buying water (OR = 3.11) and experiencing injury while collecting water (OR = 3.31), and a high normative income was associated with decreased odds of past-year water shortage (OR = 0.39).

Finally, in Gressier, an HWISE score greater than the 50th percentile was associated with high rated normative safe water, experiencing injury while collecting water, longer water collection time, and larger household size (OR = 4.33, 2.34, 1.87, 1.80, respectively). In Léogâne, an HWISE score greater than the 50th percentile was associated with experiencing past-year water shortage, injury while collecting water, high normative safe water, and longer water collection time (OR = 4.09, 3.79, 2.38, 2.31, respectively), with working (OR = 0.47) and sourcing non-drinking water from pipes were associated with decreased odds (OR = 0.27) (Table 4).

**Table 4:** Factors associated with key water-related outcomes.

	OR (95% OR CI)
Buying drinking water	
Gressier	
Age > 33	1.65 (1.02, 2.70)
Drinking piped water vs. not	0.38 (0.17, 0.80)
Low education rung vs. high rung	0.63 (0.38, 1.03)
Low income rung vs. high rung	0.60 (0.35, 1.03)
Low safe work rung vs. high rung	0.60 (0.35, 1.05)
Non-drinking pipe vs. not	3.09 (1.46, 6.98)
Water collection time > 5 minutes	1.60 (0.99, 2.62)
Léogâne	
Age > 33	1.93 (0.94, 4.09)
Drinking piped water vs. not	0.35 (0.11, 1.09)
Low education rung vs. high rung	0.49 (0.23, 1.03)
Low safe work rung vs. high rung	0.35 (0.16, 0.76)
Participant gets household water vs. does not	0.46 (0.20, 0.99)
Past-year water shortage vs. not	3.81 (1.89, 7.95)
Water collection time > 5 minutes	2.43 (1.16, 5.27)
Works for NGO vs. does not	0.38 (0.14, 1.02)
Works in healthcare vs. does not	2.40 (0.67, 10.39)
Works vs. does not work	0.30 (0.13, 0.65)
Source drinking water from pipe	
Gressier	
Buys water vs. does not	0.42 (0.19, 0.86)
Household size > 5	2.17 (1.03, 4.72)
Non-drinking piped water vs. not	50.25 (23.75, 117.22)
Works vs. does not work	1.72 (0.83, 3.72)
Léogâne	
Age > 33	0.38 (0.12, 1.10)
Buys water vs. does not	0.26 (0.08, 0.80)
Household size > 5	3.12 (1.05, 10.04)
Low safe work rung vs. high rung	0.19 (0.05, 0.63)
Non-drinking piped water vs. not	3.16 (0.70, 12.86)
Received education about health vs. has not	0.23 (0.06, 0.76)
Water collection time > 5 minutes	4.32 (1.38, 15.43)
Experienced past-year water shortage	
Gressier	
Household size > 5	2.38 (1.41, 4.05)
Low income rung vs. high rung	0.48 (0.28, 0.81)
Low safe water rung vs. high rung	2.01 (1.22, 3.36)
Stores water in house vs. not	2.12 (1.10, 4.19)
Water collection time > 5 minutes	2.20 (1.33, 3.65)
Léogâne	
Buys water vs. does not	3.11 (1.61, 6.12)
Household size > 5	1.82 (0.98, 3.42)
Injury while collecting vs. not	3.31 (1.74, 6.51)
Low income rung vs. high rung	0.39 (0.21, 0.73)
HWISE greater than 50^th^ percentile	
Gressier	
Experienced water shortage vs. never	1.56 (0.91, 2.68)
Household size > 5	1.80 (1.03, 3.18)
Injury while collecting vs. not	2.34 (1.36, 4.07)
Low safe water rung vs. high rung	4.33 (2.57, 7.39)
Water collection time > 5 minutes	1.87 (1.10, 3.18)
Léogâne	
Drinking piped water vs. not	2.89 (0.86, 10.63)
Experienced water shortage vs. never	4.09 (2.07, 8.35)
Injury while collecting vs. not	3.79 (1.88, 7.84)
Low safe water rung vs. high rung	2.38 (1.21, 4.74)
Non-drinking piped water vs. not	0.27 (0.06, 0.95)
Water collection time > 5 minutes	2.31 (1.18, 4.57)
Works vs. does not work	0.47 (0.23, 0.93)

## Discussion

This study reinforced how there are often disparities between neighboring communities despite similarities in culture and demographics. The peri-urban commune in this study, Gressier, had a majority of its inhabitants using a form of piped water (31.6%) as their primary source of drinking water, whereas in Léogâne, the urban commune, respondents largely used a bottle or sachet (44.7%). While piped water is more accessible than pumped water or buying bottled water from vendors, the quality of the water is unknown at this time despite being classified an improved water source by WHO and UNICEF [10]. Piped water has been known to cause major health impacts and often need maintenance and repair, especially in low-income countries such as Cambodia; however, it is uncertain whether community water sources, such as tube wells or boreholes are a safer alternative [17–21]. For piped water specifically, DINEPA (*Direction Nationale de l’Eau Potable et de l'Assainissement*) of the Ministry of Public Works serves as the entity in Haiti that regulates service providers as well as implements policy [22]. However, due to the unstructured network of piping in Gressier, it is unclear if it falls under DINEPA’s regulations and testing.

The results of this study showed that nearly a quarter (22.1%) of those living in Léogâne received their drinking water from a hand-pumped well. This could potentially be due to the large number of NGOs and international organizations that dug wells in Léogâne after the 2010 earthquake [23]. Additionally, those that were more likely to have a higher HWISE score also experience injury upon water collection. While we did not specifically ask how they were injured, injury during water collection is not uncommon across low and middle income countries. The long term effects of water collection can result in fatigue, soft tissue damage, musculoskeletal damage, as well as adverse effects on the skeletal system [24–25]. Those living in rural areas are often more likely to experience injury collecting water due to their higher potential for malnutrition and poor health [24–25].

Comparisons of measured socioeconomic characteristics showed broad similarities between urban and peri-urban participants, which helps minimize potential confounding relationships. However, one difference identified is that Gressier participants received general health education in significantly higher proportions than those in Léogâne. The association between water and health is a present concern to residents of Gressier and Léogâne [26] and researchers have conducted health education campaigns about the association in the past, albeit in different communities [27]. Therefore, it is possible that receiving health information changed participant water practices, though the absolute difference of 11% indicates that this may only have minimal effects.

The distributions for sources of drinking and non-drinking water were significantly different between both settings. This indicates that water quality concern, routes for intervention, and health education needs may not be comparable, despite being in the same department. Additionally, the percentage of participants who received drinking and non-drinking water from the same source differed between both settings, again indicating that water practices between the two differed in a way significant to public health practice.

Within both settings, stratified analyses showed there were no differences of median HWISE score between water sources (Table 4). This indicates that median HWISE score did not differ between water sources when adjusting for settings, drinking use, and non-drinking use. When comparing drinking and non-drinking water sources across both settings there were no differences in median HWISE scores identified (Table 3). That is, when adjusting for water source, the participant’s drinking use, non-drinking use, urban, or peri-urban exposures were not associated with differences in HWISE score.

These results may indicate that major factors of water insecurity lie in water supply system before consumers seek it, and in ways that are insensitive to small geographic variations within departments; for example, a regional drought or the lack of commercial water presence. In order to address observed water insecurity, resources may need to be dedicated to supporting more robust water supply systems, rather than intervening on individual water sources. Instead, addressing individual water sources could be the target of interventions to infectious disease and other environmental health factors.

The United Nations has proposed a series of goals that aim to lessen various global health problems, which they call the Sustainable Development Goals. The underlying focus of these goals is directly related to water and sanitation. The sixth goal on the list aims to ensure availability and sustainable management of water and sanitation worldwide [3]. Globally, more than 2 billion people live in countries that have excess water stress, which is defined as the freshwater withdrawn to total renewable freshwater resources above a threshold of 25 percent [3]. This indicates that there is a large probability that these countries will experience water scarcity in the future [3]. Clean water and proper sanitation are crucial components when trying to improve health conditions within a population. In depth understanding of contributing factors and effective strategies in place in Haiti will allow for progress made towards the achievement of the 2030 Goal 6 put in place by the United Nations.

One of the strengths of this study was the demographic similarities that were shared between participants from Léogâne and Gressier. Characteristics such as age and household size were nearly identical, which reduces the confounding effects of these characteristics. However, limitations included a small sample size between both communes and potential bias within the Léogâne data due to UF students being present during data collection. Subsequently, future studies should build upon this research to target specific areas of water hygiene and insecurity.

## Supporting information

S1 Table Participant characteristics of peri-urban and urban adults. (DOCX)

## References

[1] The World Factbook: Haiti [Internet]. Central Intelligence Agency; 2018 [cited 2019Feb14]. Available from: https://www.cia.gov/library/publications/the-world-factbook/geos/ha.html

[2] Patrick M, Berendes D, Murphy J, Bertrand F, Husain F, Handzel T. Access to safe water in rural Artibonite, Haiti 16 months after the onset of the cholera epidemic. The American journal of tropical medicine and hygiene. 2013 10 9;89(4):647–53. 10.4269/ajtmh.13-0308 24106191 PMC3795094

[3] Goal 6: Sustainable Development Knowledge Platform [Internet]. United Nations. [cited 2019Feb14]. Available from: https://sustainabledevelopment.un.org/sdg6

[4] Bertuzzo E, Finger F, Mari L, Gatto M, Rinaldo A. On the probability of extinction of the Haiti cholera epidemic. Stochastic Environmental Research and Risk Assessment. 2016 12 1;30(8):2043–55.

[5] Gelting R, Bliss K, Patrick M, Lockhart G, Handzel T. Water, sanitation and hygiene in Haiti: past, present, and future. The American journal of tropical medicine and hygiene. 2013 10 9;89(4):665–70. 10.4269/ajtmh.13-0217 24106193 PMC3795096

[6] Picard LA, Groelsema R, Buss TF. Foreign Aid and Foreign Policy: Lessons for the Next Half-century: Lessons for the Next Half-century. Routledge; 2015 1 28.

[7] Bisung E, Elliott SJ. Improvement in access to safe water, household water insecurity, and time savings: A cross-sectional retrospective study in Kenya. Social Science & Medicine. 2018 3 1;200:1–8. 29355826 10.1016/j.socscimed.2018.01.001

[8] Wutich A, Ragsdale K. Water insecurity and emotional distress: coping with supply, access, and seasonal variability of water in a Bolivian squatter settlement. Social science & medicine. 2008 12 1;67(12):2116–25. 18954928 10.1016/j.socscimed.2008.09.042

[9] Wutich A. Intrahousehold disparities in women and men's experiences of water insecurity and emotional distress in urban Bolivia. Medical anthropology quarterly. 2009 12 1;23(4):436–54. 20092053 10.1111/j.1548-1387.2009.01072.x

[10] World Health Organization & United Nations Children’s Fund. Safely managed drinking water—thematic report on drinking water 2017. Safely managed drinking water—thematic report on drinking water 2017. 2017.

[11] World Health Organization, & United Nations Children's Fund. [Internet]. Data. 2017 [cited 2019Feb14]. Available from: https://washdata.org/data

[12] Institut Haitien de Statistique et d'Informatique—IHSI. [cited 2019Feb14]. Available from: http://www.ihsi.ht/produit_demo_soc.htm

[13] Committee for the Advancement of Gressier, Haiti. [Internet]. [cited 2019Feb14]. Available from: http://www.gressier.org/about.aspx

[14] Young S.L., Collins S.M., Boateng G.B., Neilands T., Jamaluddine Z., Miller J.D., et al A protocol for the development and validation of an instrument to measure household water insecurity across cultures: the Household Water InSecurity Experiences (HWISE) scale. 2019 BMJ Open. 10.1136/bmjopen-2018-023558 PMC6340431 30782708

[15] Agresti A. An introduction to categorical data analysis. Wiley; 2018 11 13.

[16] van Buuren S, Groothuis-Oudshoorn K. MICE: Multivariate Imputation by Chained Equations in R Journal of Statistical Software, forthcoming, 2009. URL http://CRAN.R-project.org/package=mice.

[17] Cutler D, Miller G. The role of public health improvements in health advances: the twentieth-century United States. Demography. 2005 2 1;42(1):1–22. 15782893 10.1353/dem.2005.0002

[18] Watson T. Public health investments and the infant mortality gap: Evidence from federal sanitation interventions on US Indian reservations. Journal of Public Economics. 2006 9 1;90(8–9):1537–60.

[19] Country paper: Cambodia. Proceedings of the: Asian Water Development Outlook 2007 Manila: Asian Development Bank; 2007.

[20] Improving local service delivery for the MDGs in Asia: water and sanitation sector in Cambodia New York: United Nations Children’s Fund; 2009.

[21] Musemwa M. From ‘sunshine city’to a landscape of disaster: The politics of water, sanitation and disease in Harare, Zimbabwe, 1980–2009. Journal of Developing Societies. 2010 6;26(2):165–206.

[22] Direction Nationale d'Eau Potable et d'Assainissement.Retrieved from Ministry of Public Works website: https://www.dinepa.gouv.ht/

[23] Widmer JM, Weppelmann TA, Alam MT, Morrissey BD, Redden E, Rashid MH, et al Water-related infrastructure in a region of post-earthquake Haiti: High levels of fecal contamination and need for ongoing monitoring. The American journal of tropical medicine and hygiene. 2014 10 1;91(4):790–7. 10.4269/ajtmh.14-0165 25071005 PMC4183406

[24] Fry LM, Cowden JR, Watkins DW Jr, Clasen T, Mihelcic JR. Quantifying health improvements from water quantity enhancement: An engineering perspective applied to rainwater harvesting in West Africa. Environmental science & technology. 2010 11 16;44(24):9535–41. 21080624 10.1021/es100798j

[25] Graham JP, Hirai M, Kim SS. An analysis of water collection labor among women and children in 24 sub-Saharan African countries. PloS one. 2016 6 1;11(6):e0155981 10.1371/journal.pone.0155981 27248494 PMC4889070

[26] Wood EA, Chapman KS, de Rochars VM, Mckune SL. Community-based health needs assessment in Léogâne and Gressier, Haiti: six years post-earthquake. Journal of International Humanitarian Action. 2017 12 1;2(1):10.

[27] Childs L, François J, Choudhury A, Wannemuehler K, Dismer A, Hyde TB, et al Evaluation of Knowledge and Practices Regarding Cholera, Water Treatment, Hygiene, and Sanitation before and after an Oral Cholera Vaccination Campaign—Haiti, 2013–2014. The American journal of tropical medicine and hygiene. 2016 12 7;95(6):1305–13. 10.4269/ajtmh.16-0555 27799642 PMC5154444

