# Supplementary material for: Perceptions of water insecurity from urban and peri-urban Haiti: A quantitative analysis
Source: PLoS One. 2019 Apr 24;14(4):e0214789. doi: 10.1371/journal.pone.0214789 (PMC6481815; doi:10.1371/journal.pone.0214789)
Supplement: S1 Table — (DOCX) [file pone.0214789.s001.docx]

Table 1 Participant Characteristics of Peri-Urban and Urban Adults

|  | n (%) | |
| --- | --- | --- |
|  | Gressier  295 (59.1) | Léogâne  204 (40.9) |
| Socio-demographic characteristics | | |
| Age  Range  Mean (SD)  Median | (16, 85)  36.0 (14.2)  33 | (<1, 82)  35.9 (13.1)  33 |
| Household size  Range  Mean (SD)  Median | (1, 11)  5.0 (2.3)  5 | (1, 11)  5.3 (2.3)  5 |
| Median rated ladder rung  Income  Education  Safe work  Safe water | 9  5  9  5 | 8  5  8  5 |
| House type – n (%)  Owned house  Rented house  Owned apartment  Rented apartment  Other | 253 (86.1)  34 (11.6)  4 (1.4)  2(0.7)  1(0.3) | 141 (69.8)  54 (26.7)  2 (1.0)  1(0.5)  4 (2.0) |
| Occupation – n (%)  Trade/commerce  Education  Food service  Homemaker/supported  Not working  Other | 90 (30.5)  10 (3.4)  20 (6.8)  36 (12.2)  91 (30.8)  40 (13.9) | 77 (37.7)  5 (2.5)  8 (3.9)  8 (3.9)  69 (33.8)  24 (12.6) |
| Works in – n (%)  NGO sector  Healthcare sector | 39 (13.2)  31 (10.5) | 31 (15.2)  19 (9.3) |
| Received education – n (%)  About health  From work | 148 (50.2)  65 (22.0) | 80 (39.2)  36 (17.7) |
| Water characteristics | | |
| Household Water Insecurity Security Experience score  Range*  Mean (SD)  Median | (0, 72)  18.4 (15.4)  14 | (0, 88)  19.9 (16.7)  14 |
| Cost of water (HTG)  Range  Mean (SD)  Median | (0, 7000.0)  303.9 (840.0)  0 | (0, 3200.0)  287.3 (448.4)  100.0 |
| Drinking water source – n (%)  House or yard pipe  Hand pump  Protected or unprotected well  Small vendor or another person  Truck  Bottle or sachet  Rain, surface water, or other | 93 (31.6)  26 (8.8)  26 (8.8)  43 (14.6)  22 (7.5)  73 (24.8)  11 (3.7) | 19 (9.5)  44 (22.1)  18 (9.0)  24 (12.1)  4 (2.0)  89 (44.7)  1(0.5) |
| Non-drinking water source – n (%)  House or yard pipe  Hand pump  Protected or unprotected well  Small vendor or another person  Truck  Bottle or sachet  Rain, surface water, or other | 93 (31.8)  50 (17.1)  83 (28.4)  3 (1.0)  30 (10.3)  12 (4.1)  21 (7.2) | 21 (10.7)  72 (36.5)  95 (48.2)  4 (2.0)  0 (0.0)  0 (0.0)  5 (2.5) |

*Maximum possible value of 104
